# Dexamethasone protects retinal ganglion cells but not Müller glia against hyperglycemia *in vitro*

**DOI:** 10.1371/journal.pone.0207913

**Published:** 2018-11-26

**Authors:** Xandra Pereiro, Noelia Ruzafa, Arantxa Acera, Alex Fonollosa, F. David Rodriguez, Elena Vecino

**Affiliations:** 1 Department of Cell Biology and Histology, University of Basque Country UPV/EHU, Leioa, Vizcaya, Spain; 2 Servicio Oftalmología Hospital de Cruces, BioCruces, Barakaldo, Vizcaya, Spain; 3 Department of Biochemistry and Molecular Biology, University of Salamanca, Salamanca, Spain; National Eye Centre, UNITED STATES

## Abstract

Diabetic retinopathy (DR) is a common complication of diabetes, for which hyperglycemia is a major etiological factor. It is known that retinal glia (Müller cells) and retinal ganglion cells (RGCs) are affected by diabetes, and there is evidence that DR is associated with neural degeneration. Dexamethasone is a glucocorticoid used to treat many inflammatory and autoimmune conditions, including several eye diseases like DR. Thus, our goal was to study the effect of dexamethasone on the survival of RGCs and Müller glial cells isolated from rat retinas and maintained *in vitro* under hyperglycemic conditions. The behavior of primary RGC cell cultures, and of mixed RGC and Müller cell co-cultures, was studied in hyperglycemic conditions (30 mM glucose), both in the presence and absence of Dexamethasone (1 μM). RGC and Müller cell survival was evaluated, and the conditioned media of these cultures was collected to quantify the inflammatory cytokines secreted by these cells using a multiplex assay. The role of IL-1β, IL-6 and TNFα in RGC death was also evaluated by adding these cytokines to the co-cultures. RGC survival decreased significantly when these cells were grown in high glucose conditions, reaching 54% survival when they were grown alone and only 33% when co-cultured with Müller glia. The analysis of the cytokines in the conditioned media revealed an increase in IL-1β, IL-6 and TNFα under hyperglycemic conditions, which reverted to the basal concentration in co-cultures maintained in the presence of dexamethasone. Finally, when these cytokines were added to co-cultures they appeared to have a direct effect on RGC survival. Hence, these cytokines could be implicated in the death of RGCs when glucose concentrations increase and dexamethasone might protect RGCs from the cell death induced in these conditions.

## Introduction

Diabetes is a metabolic disease characterized by high glucose concentrations in the blood. One of the most common complications of this disease is diabetic retinopathy (DR), the leading cause of blindness in the population of working-age in developed countries [1]. In the symptomatic phase of DR, key clinical alterations to the vascular system occur that are relevant to the diagnosis of the disease. Indeed, for many years DR has been considered a microvascular disease, characterized by increased vascular permeability due to the breakdown of the blood-retinal barrier (BRB) [2]. Although vascular changes are a classic hallmark of this disorder, several observations suggest that microangiopathy is only one aspect of a more widespread retinal dysfunction.

The concept that neurons as well as capillaries are affected by diabetes is not new. In the early 1960s, DR was associated with the degeneration of retinal ganglion cells (RGCs) [3, 4] and indeed, apoptosis of rat retinal neurons is enhanced after chemically induced diabetes [5, 6]. In fact, diabetes-induced changes in retinal neurons and glia may precede the onset of clinically evident vascular injury. Several metabolic impairments have been implicated in the neurodegeneration associated with DR: oxidative stress, characterized by the presence of advanced glycated end products (AGEs) and nitric oxide (NO); excitotoxicity and excess glutamate receptor stimulation that provokes the uncontrolled influx of calcium into neurons; and inflammation, involving the release of chemical mediators and leukostasis [7].

Müller cells are the principal glia in the retina and they fulfill quite dynamic roles. Müller cells extend throughout the thickness of the retina, providing structural stability and maintaining close contact with the majority of retinal neurons [8, 9]. They also provide neurons with trophic factors and help to maintain retinal homeostasis, potentially promoting cell survival and repair [10, 11]. Although the physiology of these cells was previously thought to be rather simple, studies over the past 2 decades have revealed that Müller cells express a diversity of ion channels and transporters, that they release a range of cytokines and survival factors, and that they express receptors for numerous neurotransmitters and growth factors [12, 13]. In fact, it has been shown that under hyperglycemic conditions, Müller glial cells contribute to the development and progression of diabetes by enhancing caspase-1/IL-1β signaling and mitochondrial stress [14, 15]. In addition, Müller cells markedly up regulate their expression of glial fibrillary acidic protein (GFAP) early in the course of DR [16], a non-specific response to the pathophysiological conditions [17].

Dexamethasone (DEX) is a synthetic corticosteroid that displays anti-inflammatory and immunosuppressive activity. It was first used for an eye-related disease in 1974, when intravitreal (IVT) injection was employed to treat experimentally induced *Pseudomonas* endophthalmitis in rabbits [18]. Nowadays, clinical treatment of eye-related conditions with DEX usually involves administration of slow-release intravitreal implants. These are mostly used to treat macular edema (ME) and diabetic ME (DME), producing favorable results on visual acuity (VA) [19–21], as well as in diabetic patients [22–24]. Furthermore, a recent long-term study into the use of DEX implants showed that it has the potential to not only delay DR progression but also, it may reduce DR severity over 24 months [25]. Nevertheless, the mechanisms underlying the effects of this glucocorticoid are not entirely clear. However, IVT DEX injection is known to regulate immune responses and to diminish vascular damage by decreasing cell permeability in rat models of streptozotocin-induced diabetes [26, 27], possibly acting through the TNFα pathway [28].

*In vitro* studies have helped to characterize the retinal damage associated with diabetes. Cell culture models represent simplified systems to assess the effects of different toxic factors in the diabetic milieu, such as excess glucose, glutamate and AGEs. We previously showed that primary cultures of adult rat Müller glia and RGCs express the same trophic factors and receptors as they do *in vivo* [29]. Moreover, Müller glia cells contact RGCs in co-culture and the factors secreted by Müller glia into the medium, can protect pig RGCs from death [30, 31]. Studies on endothelial and pericyte cell cultures have helped highlight the mechanisms underlying the cell damage in diabetes, yet studies on retina neuroglial cultures have been scarce [32]. Thus, here we set out to study the effect of high levels of glucose, on RGC survival. In order to identify the role of Müller glia and their relationship to RGCs, we compared pure RGC cultures with RGC-Müller glia co-cultures in different glucose concentrations *in vitro*. Moreover, to better understand the benefits that DEX produces in DR, we also examined its effect on purified rat RGCs, as well as on RGCs co-cultured with Müller glia maintained under hyperglycemic conditions. The role of inflammation in this process was also assessed by measuring the cytokines that accumulate in the medium of these cultures, thereby identifying cytokines that might protect RGCs from the toxic effects of hyperglycemia.

## Materials and methods

### Retinal cell cultures

This study was carried out in strict accordance with the recommendations in the Guide for the Care and Use of Laboratory Animals. The experimental protocol met European (2010/63/UE) and Spanish (RD53/2013) regulations for the protection of experimental animals, and it was approved by the Ethical Committee for Animal Welfare of the University of Basque country. RGCs and Müller glia were isolated from adult female Sprague-Dawley rats, humanely sacrificed by exposure to CO_2_, establishing two types of culture: (1) Pure RGC cultures; and (2) co-culture of RGCs and Müller glial cells. The retinas were dissected and dissociated enzymatically with papain (Worthington Papain Dissociation kit, Worthington Biochemical Lakewood, NJ, USA) for 90 (RGCs) or 30 min (co-cultures), according to the manufacturer’s instructions. The dissociated cells were recovered by centrifugation and the RGC cultures were prepared as described previously [31]. Briefly, the dissociated cells were passed through an ovomucoid inhibitor-albumin gradient, where more RGCs than Müller cells pass due to their larger size (this step was excluded when preparing the co-cultures). While this gradient does not purify RGCs to homogeneity, there is only minimal contamination of other cells (data not shown). After purification, the cells were seeded on 13 mm diameter poly-L-lysine (10 g/ml: Sigma–Aldrich, St. Louis, MO, USA) and laminin (10 g/ml: Sigma–Aldrich, St. Louis, MO, USA) coated coverslips in 24-well plates. The RGCs were seeded at 10^5^ viable cells per well and the co-culture of RGCs and Müller glia were established at 6x10^6^ viable cells per well (as determined by trypan blue test).

Different media were used for each culture type, containing 1% L-glutamine (2 mM: Life Technologies, Carlsbad, CA, USA) and 0.1% gentamicin (50 mg/ml: Life Technologies, Carlsbad, CA, USA): Neurobasal-A medium supplemented with 2% B27 (Life Technologies, Carlsbad, CA, USA) for RGC cultures; and B27 supplemented Neurobasal-A medium with 10% FBS (Fetal Bovine Serum: Life Technologies, Carlsbad, CA, USA) for RGC and Müller mixed cell cultures. In addition, each culture type was maintained under distinct conditions: i) Control (untreated); ii) 1 μM DEX (Sigma Aldrich, Saint Louise, MO, USA); iii) 10 mM Glucose; iv) 30 mM Glucose; and v) 30 mM Glucose + 1μM DEX. The different conditions were employed from the start of the culture and the medium was changed every 48 hours. At least 4 replicates of each culture type were performed in triplicate. In addition, a further control involved maintaining the cells in the presence of 30 mM mannitol to demonstrate that the osmolarity of the glucose solution did not affect the results. Exposure to mannitol induced no significant changes (results not shown).

### Immunocytochemistry

After 6 days in culture, the cells were fixed in cold methanol and washed with PBS (phosphate buffered saline, pH 7.0). After blocking non-specific antigens with blocking buffer (3% BSA and 0.1% Triton X-100 in PBS), the cells were incubated with the following primary antibodies at a dilution of 1:2,000: a mouse anti-βIII-tubulin antibody (Promega Madison, WI, USA) as a specific RGC marker and a rabbit anti-Vimentin antiserum (Abcam, Cambridge, England) as a specific marker of Müller cells. After washing the cells, antibody binding was detected with an anti-mouse Alexa Fluor 488 and an anti-rabbit Alexa Fluor 555 (Life Technologies, Carlsbad, AC, USA) secondary antibodies, diluted 1:1,000. In addition, the cell nuclei were labeled with DAPI (Life Technologies, Carlsbad, AC, USA) at a dilution of 1:10,000.

### Cell quantification

The same number of cells were seeded per plate for each condition and in each experiment, and the cultures were analyzed on an epifluorescence microscope (Zeiss, Jena, Germany) coupled to a digital camera (Zeiss Axiocam MRM, Zeiss, Jena, Germany). The total number of RGCs and Müller cells per coverslip was quantified using the specific markers indicated. The Müller cell counts were normalized to the number of cells grown in the control conditions, considered as 100% survival. The RGCs and Müller cells in co-cultures were counted in images obtained with a filter specific for only one of the antibody markers. However, since there may be an overlap with vimentin stained cells, the semi-automatic Zen software (Zeiss, Jena, Germany) was used to count the number of nuclei stained with DAPI, taking into consideration the limits of the axis of the nuclei of Müller cells to achieve more accurate measurements. For that purpose, we used a specific macro designed to specifically measure the limits of the axes (50–200 μm), which was corrected manually for each image. At least 4 complete coverslips and 3 independent experiments were analyzed for each experimental condition.

### Multiplex cytokine assays

After 6 days in culture, conditioned medium (supernatant) was collected for each experimental condition, filtered and the cytokines present were quantified with a Q-Plex multiplex enzyme-linked immunosorbent assay (Q-Plex Rat Cytokine—Inflammation (9-Plex): Quansys Bioscience, Logan, UT, USA). The presence of 9 cytokines in 150 μl of each sample was assessed in a 98-well Q-Plex plate according to the manufacturer´s instructions: IL-1α, IL-1β, IL-2, IL-4, IL-6, IL-10, IL-12, IFNγ and TNFα. The standards were measured in duplicate and the cytokine concentrations were calculated using a standard curve. Four replicates were assessed for each sample and arithmetic averages were calculated. The data were normalized per 10,000 cells.

### Cell cultures analysis adding IL-1β, IL-6 and TNFα

The direct effects of the IL-1β, IL-6 and TNFα cytokines were assessed in RGC-Müller cells co-cultures. We used the combination of these cytokines because they were found to be overexpressed in the supernatant of the cultures treated with 30 mM glucose. Six different conditions were analyzed: (1) Control (Neurobasal-A medium supplemented with 2% B27 and 10% FBS); (2) 1 μM DEX; (3) a mix of the three cytokines (IL-1β, IL-6 and TNFα: Sigma-Aldrich, St. Louis, MO, USA) at 10 ng/ml, as suggested by the supplier in the data sheet; (4) the cytokine mix at the concentrations in the conditioned medium identified by the multiplex cytokine assay: 16.75 pg/ml for IL-1β, 435 pg/ml for IL-6 and 32.1 pg/ml for TNFα; and (5) and (6) the mixtures of cytokines at both concentrations, respectively plus 1 μM DEX. The cytokines were added to the culture from the fourth (when the culture was stabilized) to the sixth day of culture (a 48 hour treatment), and after the sixth day the culture was fixed, immunostained and the cells were quantified as indicated above.

### Statistical analysis

Statistical analyses were carried out using the IBM SPSS Statistical software (v. 21.0), calculating the mean and standard error for each condition. The data from the different experimental conditions were compared using an analysis of variance (ANOVA), followed by the Tukey or Games-Howell test depending on the homogeneity of the variances. Differences were considered significant for all tests at p< 0.05.

## Results

### RGC cultures

**Fig 1:**
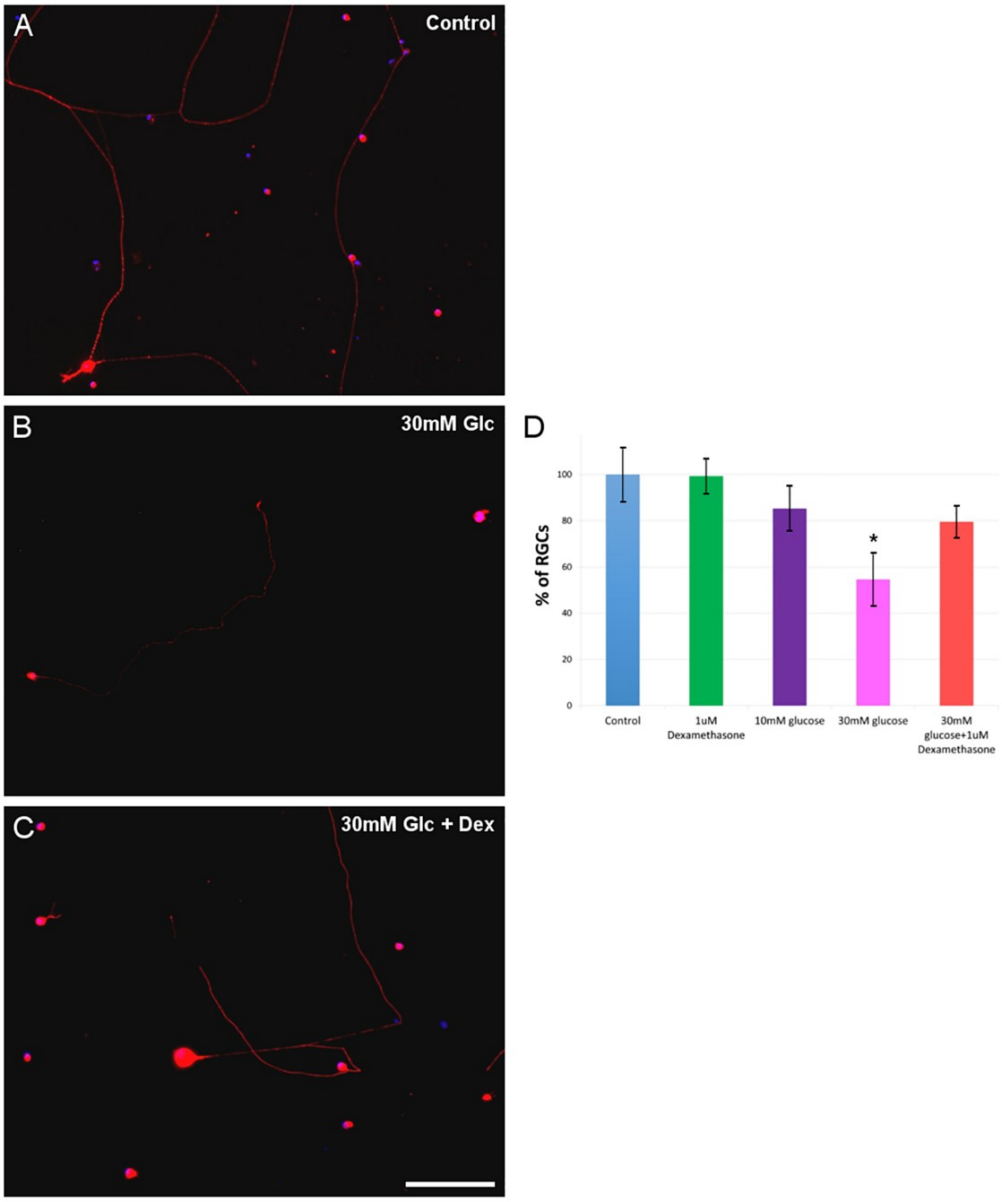
RGC cultures. Images of RGC cultures in control conditions (A), in the presence of 30 mM glucose (B) or 30 mM glucose plus 1μM dexamethasone (C). The RGCs were labeled with an antibody against Beta III-Tubulin (red) and with DAPI for nuclear counterstaining. (D) Percentage of RGCs surviving in the different conditions. A loss of RGC survival is evident in the presence of 30 mM glucose, while the presence of 1 μM dexamethasone rescued the RGCs from death, reverting survival to the basal values. The data are presented as the mean + SEM. Scale bar = 100 μm, *p<0.05.

To analyze how hyperglycemia affects RGC survival, the RGCs in primary cultures were counted in the control conditions and following the different experimental manipulations (Fig 1). When considered relative to the controls (considered as 100% survival: Table 1, see Table 1 in S1 File for the raw data), maintaining RGCs in the presence of 1 μM DEX or 10 mM glucose had no significant effect on cell survival (p>0.05). By contrast, RGC survival diminished significantly relative to the controls in the presence of 30 mM glucose (54.64 ± 6.74%, p<0.05). Interestingly, this impairment in RGC survival in the presence of 30 mM glucose was partially recovered by adding 1 μM DEX to the cell cultures (79.53 ± 12.24% cell survival), a survival rate that was not significantly different to that recorded in the controls. However, when RGC survival in the presence of 30 mM glucose was compared with that in the presence of 30 mM glucose and DEX, there was no clear difference in survival (p = 0.477, see S2 File for statistical analysis: Fig 1). Hence, DEX appeared to at least partially ameliorate the effect of high glucose on RGC survival in culture.

**Table 1:** Survival of RGCs in pure culture of RGCs.

EXPERIMENTAL CONDITION	% of RGCs	n	p value compared to the control
Control	100 ± 11.46	12	-
1 μM Dexamethasone	99.29 ± 7.54	12	1.000
10 mM Glucose	85.30 ± 11.51	12	0.846
30 mM Glucose	54.64 ± 6.74	10	**0.032**
30 mM Glucose + 1 μM Dexamethasone	79.53 ± 12.24	12	0.618

The data are expressed as the proportion of surviving cells (mean + SEM), evaluated by ANOVA followed by the Tukey or Games-Howell test depending on the homogeneity of variances.

### Co-cultures

**Fig 2:**
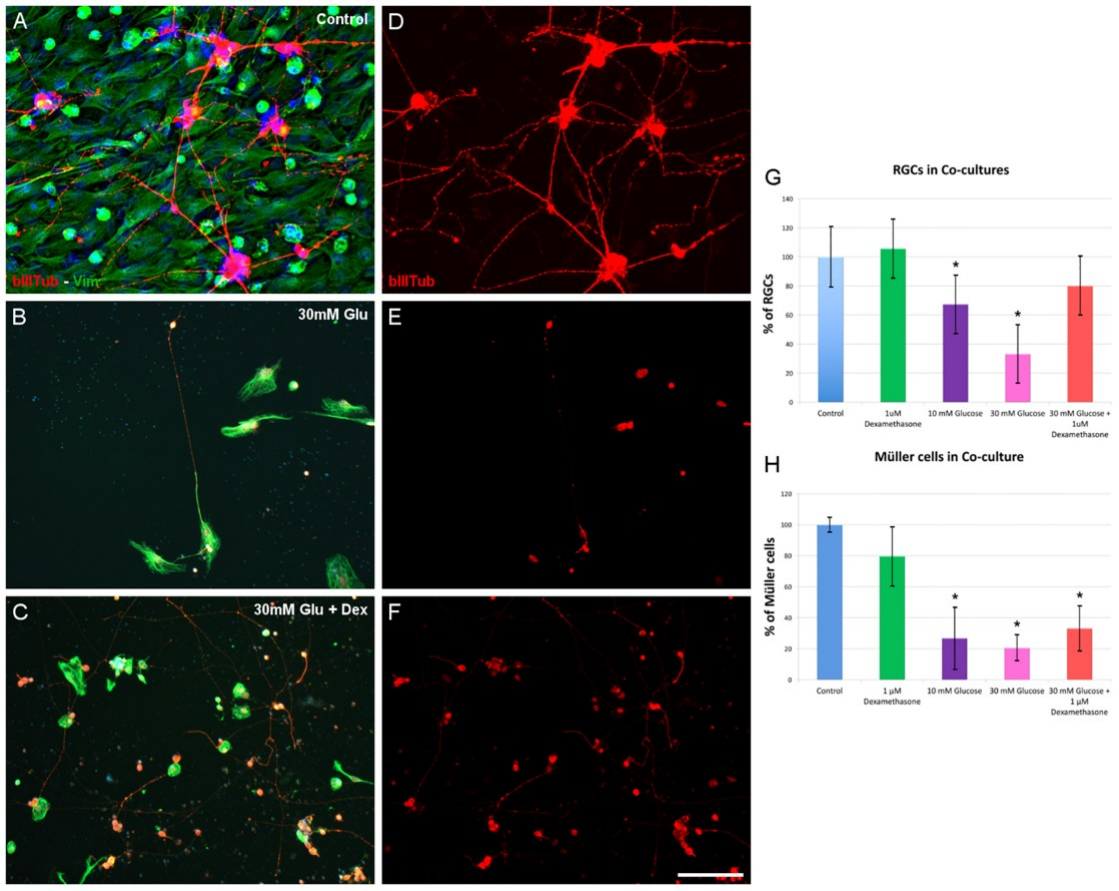
Co-cultures of RGCs and Müller cells. RGCs were labeled with an antibody against Beta III-Tubulin (red) and Müller cells with a Vimentin antibody (green). Note the increased survival of RGCs in the presence of dexamethasone when co-cultures are maintained in the presence of 30 mM Glucose (compare B and E to C and F). (G) RGC survival in the different conditions. RGC survival decreases on exposure to 10 mM (67.35 ± 9.41%, *p<0.05) or 30 mM glucose (33.10 ± 4.03%, *p<0.05), while the presence of 1 μM Dexamethasone enhances the survival of RGCs, reverting it to control levels. (H) The proportion of Müller cells was significantly affected by exposure to 30 mM glucose (19.52 ± 4.84%,*p<0.05), yet this decrease in Müller cell survival was not reversed in the presence of 1 μM dexamethasone. The data represent the mean ± SEM. Scale bar = 100 μm, *p<0.05.

Given the supportive and neuroprotective properties of Müller cells, we assessed the effects of glucose and DEX on the survival of RGCs when they were co-cultured with Müller cells. Taking the control cultures as 100%, 67.35 ± 9.41% (p<0.05) of the RGCs survived 6 days in co-culture in the presence of 10 mM glucose, and this figure dropped to 33.10 ± 4.03 (p<0.05) in the presence of 30 mM glucose (Table 2, see Table 2 in S1 File for the raw data). However, this significant decrease in cell survival was rescued by adding 1 μM DEX to the cultures, in the presence of which the survival of RGCs was no different to that in the control cultures. Indeed, RGC survival in the presence of 30 mM glucose improved significantly when DEX was added to the co-cultures (p = 0.001: Fig 2, see S3 File for the statistical analysis).

**Table 2:** Survival of RGCs in co-culture.

EXPERIMENTAL CONDITION	% of RGCs	n	p value compared to control
Control	100 ± 7.37	12	-
1 μM Dexamethasone	105.65 ± 8.22	10	0.986
10mM Glucose	67.35 ± 9.41	10	**0.040**
30 mM Glucose	33.10 ± 4.03	11	**0.000**
30 mM Glucose + 1 μM Dexamethasone	80.04 ± 9.66	11	0.366

The data are expressed as the percentage of surviving cells (mean ± SEM), evaluated using ANOVA followed by the Tukey or Games-Howell test depending on the homogeneity of variances.

Indeed, the survival of Müller cells decreased significantly in the co-cultures in the presence of both 10 mM (26.15 ± 8.35%, p<0.05) and 30 mM glucose (19.52 ± 4.84% relative to the controls, p<0.05: Table 3 and S1 File for the raw data). However, dexamethasone (1 μM) had no significant effect on the survival of Müller cells in high glucose media (36.82 ± 8.01%), with no differences in cell number relative to the co-cultures maintained in the presence of 30 mM glucose alone (p = 0.384) and with significantly fewer Müller cells than in the control conditions (p<0.05: Fig 2, see S4 File for the statistical analysis).

**Table 3:** Survival of Müller cells in co-culture.

EXPERIMENTAL CONDITION	% of Müller cells	n	p value compared to control
Control	100 ± 4.78	10	-
1 μM Dexamethasone	78.84 ± 10.58	9	0.408
10mM Glucose	26.15 ± 8.35	8	**0.000**
30 mM Glucose	19.52 ± 4.84	10	**0.000**
30 mM Glucose + 1 μM Dexamethasone	36.82 ± 8.01	10	**0.000**

The data are expressed as the relative cell survival (mean + SEM), analyzed by ANOVA followed by the Tukey or Games-Howell test depending on the homogeneity of variances.

### Cytokine assay

The amounts of the inflammatory cytokines in the conditioned media obtained from the two types of cultures were analyzed for each experimental condition, quantifying the following cytokines: IL-1α, IL-1β, IL-2, IL-4, IL-6, IL-10, IL-12, IFNγ, and TNFα (Table 4, see Table 4 in S1 File for raw data). With pure RGC cultures, no reliable data were obtained due to the small number of cells in this type of culture (approx. 100 cells per coverslip in the control conditions).

**Table 4:** Concentration of cytokines in the different conditioned media obtained from RGC and Müller cell co-cultures (data normalized per 10,000 cells).

Cytokine	Control	1 μM Dexamethasone	10 mM Glucose	30 mM Glucose	30 mM Glucose + 1 μM Dexamethasone
**IL-1α**	40.55±17.36	6.31±6.31	0±0	18.77±11.06	50.34±24.32
**IL-1β**	0±0	0±0	0±0	61.89±8.23	0±0
**IL-2**	0±0	0±0	0±0	45.74±37.57	45.86±34.16
**IL-4**	13.85±0.49	3.44±2.89	3.77±2.57	2.99±1.88	2.56±2.56
**IL-6**	589.73±31.49	178.92±45.77	764.61±76.95	1209.22±250.89	510.17±96.18
**IL-10**	5.89±0.18	4.23±0.45	5.89±0.71	11.45±0.97	9.30±0.53
**IL-12**	15.78±9.26	13.09±7.80	16.40±9.91	14.38±14.17	30.08±9.66
**IFNγ**	69.39±24.18	97.35±5.61	146.95±13.34	15.15±15.15	146.85±52.15
**TNFα**	65.60±10.75	18.83±6.66	62.55±4.01	118.61±7.78	59.07±13.08

The data are expressed as pg/ml (mean + SEM).

**Fig 3:**
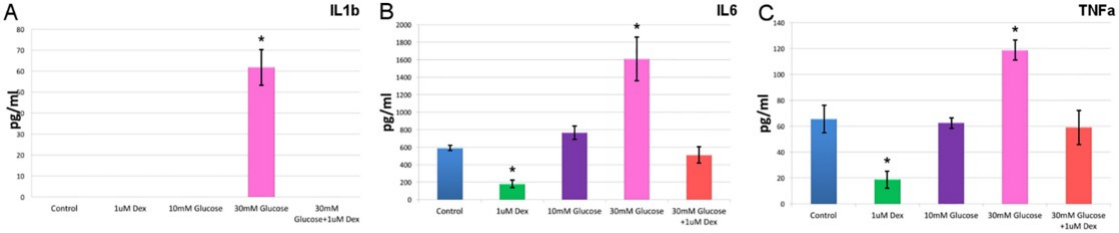
IL-1β, IL-6 and TNFα levels in the co-cultures. The changes in the three most significant cytokines of the 9 analyzed are shown: the IL-1β (A), IL-6 (B) and TNFα (C) in the conditioned medium of the RGC and Müller cell co-cultures under different conditions. The amounts of these cytokines increases in the presence of 30 mM glucose, yet they revert to the control levels when dexamethasone (1 μm) was added (*p<0.05).

In the co-cultures, the presence of 30 mM glucose provoked a significant increase in three of the nine cytokines evaluated, with the concentration of IL-6 increasing 172% (2.72 fold, 1609.22 ± 250.89 pg/ml, p<0.05) relative to the control condition (589.73 ± 31.49 pg/ml), that of TNFα increasing 81.5% (1.80 fold, 118.61 ± 7.78 pg/ml, p<0.05) relative to the control (65.60 ± 10.75 pg/ml), and while IL-1β was undetectable in the control conditions, its concentration increased to 61.89 ± 8.23 pg/ml (p<0.05) in these high glucose conditions. When DEX was added to the cultures maintained in the presence of 30 mM glucose, the concentration of these three cytokines (IL-6, TNFα and IL-1β) reverted to the control values (Fig 3).

IL-2 was also undetectable in control conditions, yet the concentration increased to 45.74 ± 37.57 pg/ml in the presence of high glucose. Similarly, the small quantities of IL-10 that were detected in control cultures (5.89 ± 0.18 pg/ml) also increased significantly in the presence of high glucose (11.55 ± 0.97 pg/ml, p<0.05). Importantly, DEX did not alter the accumulation of these two cytokines in the presence of 30 mM glucose. No significant changes were evident in the accumulation of the other cytokines analyzed (IL-1α, IL-4, IL-12 and IFNγ) between any of the different experimental conditions relative to the controls (Table 1, see S5 File for statistical analysis).

### Effect of IL-1β, IL-6 and TNFα on co-cultures

**Fig 4:**
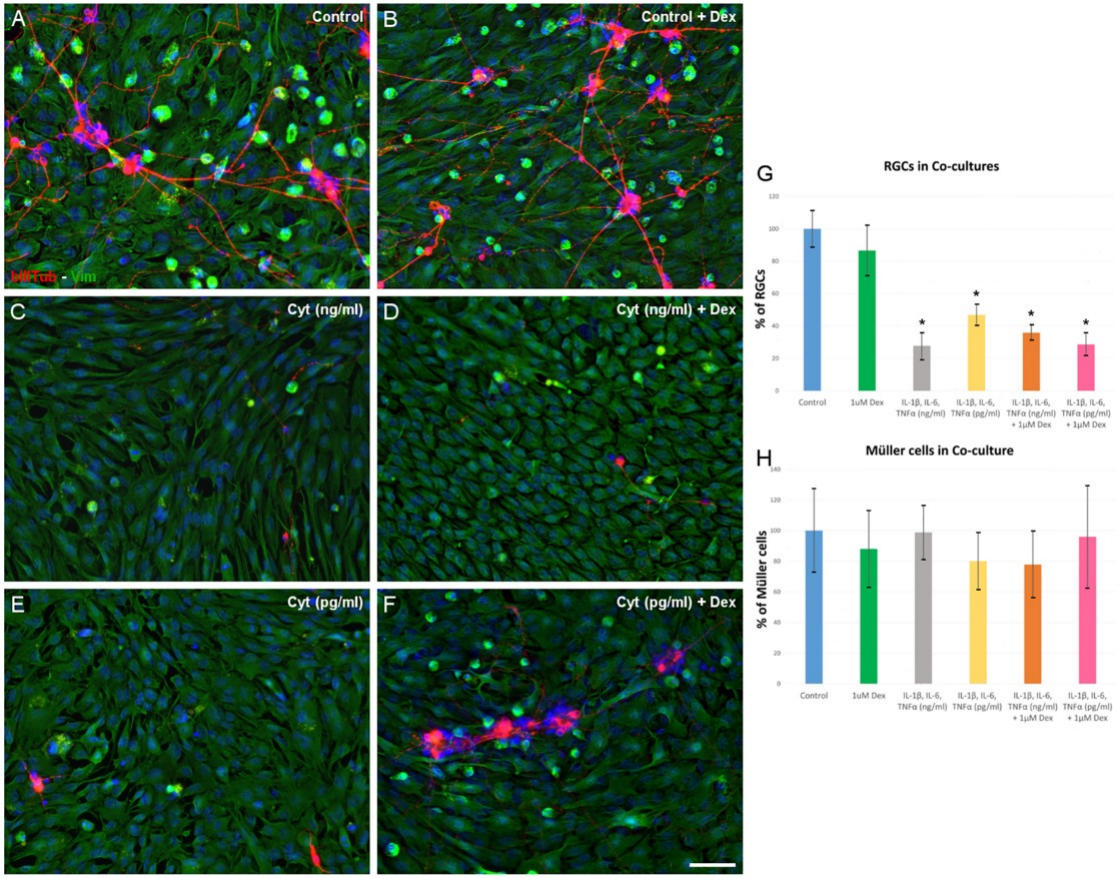
Effect of IL-1β, IL-6 and TNFα on the co-cultures. The RGCs were labeled with an antibody against Beta III-Tubulin (red) and the Müller cells with an antibody against Vimentin (green). Note the decreased RGC survival in the presence of the cytokines at both concentrations (C, E) and in combination with dexamethasone (D, F). (G) The percentage of RGCs surviving in the different conditions. (H) There were no changes in the proportion of Müller cells under each of the experimental conditions. The data represent the mean ± SEM: *p<0.05. Scale bar = 100 μm.

The effect of IL-1β, IL-6 and TNFα on co-cultures was analyzed to confirm the role of these cytokines in RGC death. In addition, the effect of DEX in combination with these three cytokines was also assessed (Fig 4).

With the control cultures as a reference for 100% survival (Table 5, see Table 5 in S1 File for the raw data), 27.78 ± 5.96% (p<0.05) of the RGCs survive after 6 days in culture in the presence of the three cytokines (10 ng/ml) for 48 hours. In the presence of lower concentrations of IL-1β, IL-6 and TNFα (pg/ml) for the same period, 46.89 ± 2.88% of RGCs survive (p<0.05). However, the presence of DEX at either concentration of the cytokines failed to rescue RGCs from death, and the survival rates of RGCs in the presence of DEX were 35.91 ± 5.81% and 28.62 ± 5.13% for the ng/ml and pg/ml concentrations of cytokines, respectively. These rates were significantly lower than in the control conditions (p<0.05) but they were not significantly different to when these cultures were maintained in the presence of these cytokines alone (p = 0.993 and p = 0.811 for the ng/ml and pg/ml cytokine concentrations, respectively: see S6 File for the statistical analysis).

**Table 5:** Survival of RGCs in co-culture in presence of cytokines.

EXPERIMENTAL CONDITION	% of RGCs	n	p value compared to control
Control	100 ± 11.56	3	-
1μM Dex	86.62 ± 18.22	5	0.947
IL-1β, IL-6, TNFα (ng/ml)	27.78 ± 5.96	4	**0.003**
IL-1β, IL-6, TNFα (ng/ml) + 1μM Dex	35.91 ± 5.81	4	**0.009**
IL-1β, IL-6, TNFα (pg/ml)	46.89 ± 2.88	4	**0.036**
IL-1β, IL-6, TNFα (pg/ml) + 1μM Dex	28.62 ± 5.13	4	**0.003**

The data are expressed as the percentage of surviving cells (mean ± SEM), assessed by ANOVA followed by the Tukey or Games-Howell test depending on the homogeneity of variances.

Müller cell survival in the co-cultures was not affected by the presence of either concentration of the cytokines, with 98.88 ± 17.68% Müller cell survival in the presence of ng/ml cytokine concentrations and 80.11 ± 18.49% with the pg/ml concentrations of cytokines, showing no differences relative to the controls (Table 6, see Table 6 in S1 File for raw data). Similarly, DEX (1 μM) had no significant effect on the survival of Müller cells at either cytokine concentration: 77.84 ± 21.83% Müller cell survival in the presence of ng/ml cytokines; and 96.02 ± 33.59% in pg/ml concentrations of the cytokines (p = 0.972 by ANOVA, meaning that there are no significant differences between the groups: see S7 File for the statistical analysis).

**Table 6:** Survival of Müller cells in co-culture in presence of cytokines.

EXPERIMENTAL CONDITION	% of Müller cells	n
Control	100 ± 27.63	4
1μM Dex	88.08 ± 25.06	3
IL-1β, IL-6, TNFα (ng/ml)	98.88 ± 17.68	4
IL-1β, IL-6, TNFα (ng/ml) + 1μM Dex	77.84 ± 21.83	3
IL-1β, IL-6, TNFα (pg/ml)	80.11 ± 18.49	4
IL-1β, IL-6, TNFα (pg/ml) + 1μM Dex	96.02 ± 33.59	3

The data represent the percentage of surviving Müller cells (mean + SEM).

## Discussion

Hyperglycemia is considered a major factor in the etiology of DR. Like the brain, the retina primarily utilizes glucose to synthesize ATP and retinal neurons preferentially metabolize glucose rather than lactate *in vitro* [33]. While RGCs obtain some glucose directly, they also obtain glucose through astrocytes [34] or Müller glial cells [35].

As shown here, retinal neurons are particularly susceptible to long-term hyperglycemia. In our experimental set-up, a high concentration of glucose (30 mM) induces RGC death in culture, an effect that is partially reversed by DEX, raising the possibility that RGCs themselves could secrete pro-inflammatory agents that act as autocrine effectors and that possibly induce cell death. We analyzed the secretion of pro-inflammatory cytokines by RGCs, yet the test used was not sufficiently sensitive given the small number of pure RGCs that can be cultured. Neurons are typically examined as targets for cytokine signaling, with various cytokine receptors expressed on neural membranes [34, 35]. However, neurons and specifically RGCs, are also a source of releasable cytokines including: Interleukin 3 (IL-3), TNF-α, CXCL9, VEGF, L-selectin, IL-4, GM-CSF, IL-10, IL-1Rα, MIP and CCL20 [36–38]. Indeed, high levels of glucose activate the overexpression of pro-inflammatory cytokines (IL-1β and IL-18) by RGCs [39], and the expression of TLR-2 and TLR-4 by RGCs, enhancing the secretion of pro-inflammatory factors [40–42]. Although DEX partially rescues RGCs exposed high glucose and we believe that inflammatory pathways could be implicated in this effect, it is possible that other mechanisms are also at play. Many factors could be involved in the death of RGCs associated with hyperglycemia and indeed, such a high-glucose environment could enhance oxidative stress and mitochondrial dysfunction, thereby accelerating the apoptosis of RGCs via caspase-3 activation [39, 41, 43]. It is noteworthy that because of the high-energy demand of RGCs, they have large numbers of mitochondria [44, 45], and consequently, it is not surprising that they are particularly vulnerable to any mitochondrial dysfunction in DR. In addition, the expression of the high mobility group box 1 (HMGB-1), a chromatin protein that can promote angiogenesis and induce inflammation, may also be implicated in hyperglycemic RGC death [46].

RGCs are more adversely affected by high glucose concentrations (30 mM) when they are co-cultured with Müller cells than when growing in pure culture. Furthermore, at 10 mM glucose RGCs are significantly affected when they are co-cultured with Müller cells, with the survival of the latter also affected by high glucose conditions. Müller cell death following exposure to high glucose conditions (25–30 mM glucose) has been described elsewhere [47, 48], and it has potentially been related to AKT inhibition [49]. Indeed, when rat retinal Müller glia were exposed to 25 mM glucose for 72 h culture, Akt was inactivated and apoptosis induced. Moreover, hyperglycemia stimulates GAPDH accumulation in the nucleus of retinal Müller cells, in association with the apoptosis of these cells. [47, 50]. It is possible that Müller cell death could also affect RGCs, accentuating the deleterious effect of glucose on RGCs. Moreover, Müller cell gliosis could damage RGCs under conditions of stress. Gliosis of Müller cells can have both cytoprotective and cytotoxic effects on retinal neurons depending on the extent of glial activation [8]. It is already known that diabetes induces abnormalities in retinal Müller cells, including increased GFAP expression, reduced glutamine synthetase and decreased glutamate transporter activity [51]. It is well established that Müller cells become activated in DR [52–54] but nevertheless, the contribution of Müller cells to DR remains unclear [47].

RGC death caused by exposure to high glucose might be provoked by the up-regulation of secreted pro-inflammatory factors [40, 46] and several studies confirm the relevance of Müller glia in the provision of inflammatory factors after activation, such as cytokines. Müller glia are the main source of many cytokines and other inflammatory factors in the gliotic retina *in vitro* [55], which may serve to protect Müller cells from diabetic insult, and consequently, retinal neurons. The damage produce by these factors in DR might be only a secondary effect [56]. In DR, Müller cells exhibit a reactive phenotype characterized by the activation of inflammatory-related genes [8]. The proinflammatory response of Müller glia has been associated to the activation of the receptor for advanced glycation end-products (RAGE) and its ligand S100B, which stimulate cytokine production through MAPK signaling pathways [57]. The accumulation of these inflammatory mediators is thought to enhance early neuronal cell death in the retina [55], which would explain the contribution of Müller cells to the decrease in RGC survival in our co-cultures.

Inflammation is considered to play a critical role in the progression of diabetic complications, including DR [37]. Glucocorticoids like DEX are powerful anti-inflammatory compounds. They are steroid hormones that cross the cell membrane and interact with intracellular glucocorticoid receptors (GCRs). The distribution of GCRs in the retina is highly conserved and as they are almost exclusively restricted to Müller glia, and GCRs might enhance survival indirectly through these glial cells [58, 59]. The data obtained here show that when RGCs are exposed to high glucose, more RGCs die when they are in contact with Müller glia than when they grow alone. However, DEX rescues these cells in both situations, which indicates it has a direct effect on RGCs. Glucocorticoids like DEX can induce endotoxin tolerance, acting as negative regulators of TLR4 signaling, and efficiently downregulating the production of pro-inflammatory cytokines [45].

The anti-inflammatory properties of DEX may rescue RGCs from death and thus, our results suggest that inflammation plays a critical role in the RGC death provoked by high glucose concentrations *in vitro*. To determine the effect of DEX on RGC survival in such high glucose conditions and its critical influence on inflammation in DR, the implication of cytokines in the inflammatory response caused by hyperglycemia was analyzed in conditioned medium. The levels of IL-1β, IL-6 and TNFα increase significantly in co-cultures maintained in the presence of high glucose, while DEX reverts the concentration of these cytokines to basal values. Hence, these cytokines may be involved in RGC death, since RGC survival decreases when the concentration of these cytokines augments.

When we cultured RGCs and Müller cells with the three cytokines and in the presence of DEX, IL-1β, IL-6 and TNFα appear to be directly involved in RGC death. However, DEX could not rescue RGCs maintained in the presence of the cytokines suggesting it has no effect once they are secreted into the medium. Thus, DEX might be implicated in dampening the release or expression of these cytokines by Müller glia and RGCs. Glucocorticoids like DEX, can exert multiple anti-inflammatory effects, even directly interacting with the transcriptional machinery through the TLRs expressed by Müller cells [60] and RGCs [39]. TLR signaling pathways may be important targets for DEX [61] and thus, DEX could activate the TLR signaling that inhibits the inflammatory response.

IL-1β, IL-6 and TNFα are pro-inflammatory cytokines, and IL-6 is a potential mediator of intraocular inflammation [38], inducing numerous physiological and immune responses [52]. Müller glia cells release several inflammatory factors and cytokines [55], and some cytokines are even known to stimulate the production of other cytokines by Müller glia [62], including IL-6 [62], IL-1β [53] and TNFα [54] in response to different stressors. Moreover, Müller cells are a major source of retinal IL-1β [19, 20, 63, 64] and they also secrete TNFα, facilitating the apoptotic death of RGCs in response to damage [65]. Together, these findings suggests that in hyperglycemic conditions, Müller cells could enhance RGC death in co-cultures by releasing these cytokines, while DEX can reverse this effect by recovering basal cytokine levels. High glucose conditions also increase the concentrations of IL-2 and IL-10, yet DEX does not decrease the production of these cytokines. Increased IL-2 has been seen in experimental models of DR [65], while IL-10 is an anti-inflammatory cytokine [66], which may be related to protection against the development of late diabetic complications [67]. Indeed, DEX induces the release of anti-inflammatory cytokines like IL-10 less than that of pro-inflammatory cytokines [68].

The death of Müller cells in hyperglycemic conditions may be related to other mechanisms not assessed here. This would be consistent with the failure of DEX to rescue these cells and their insensitivity to exogenous cytokine addition. Such effects may be associated with perturbations to essential functions, such as neurotransmitter recycling, the control of glutamate toxicity, a redistribution of ions or the regulation of metabolic pathways, among others [10, 56]. For example, the expression of Kir4.1 (an inwardly rectifying potassium channel) is altered by TNF-α in the Müller cells of diabetic patients [67]. Other cytokines not considered here could also alter adaptor molecules like Act 1, thereby provoking damage [68]. Also, in a context of diabetes-induced retinal gliosis, long non-coding RNAs affect Müller cell viability and proliferation [69]. Indeed, oxidative stress suppresses glutamine synthetase activity in diabetic conditions, mediated by the thioredoxin-interacting protein, TXNIP [70]. Additionally, Glutaredoxin, an enzyme that catalyzes the deglutationation of key targets like IKK (a kinase that activates NF-κβ receptors), appears to be associated with elevated IL-6 secretion in vitro [71].

Our findings may have therapeutic implications. DME is the main cause of vision loss in patients with DR [69] and the most common first line therapy for DME is IVT injection of anti-VEGF agents [70, 71]. Another therapeutic option for DME is the injection of Ozurdex, an DEX slow release IVT implant that administers this drug over 4 to 6 months. Thus, DEX may also protect RGCs in the context of DR when used in a slow release formula.

**Fig 5:**
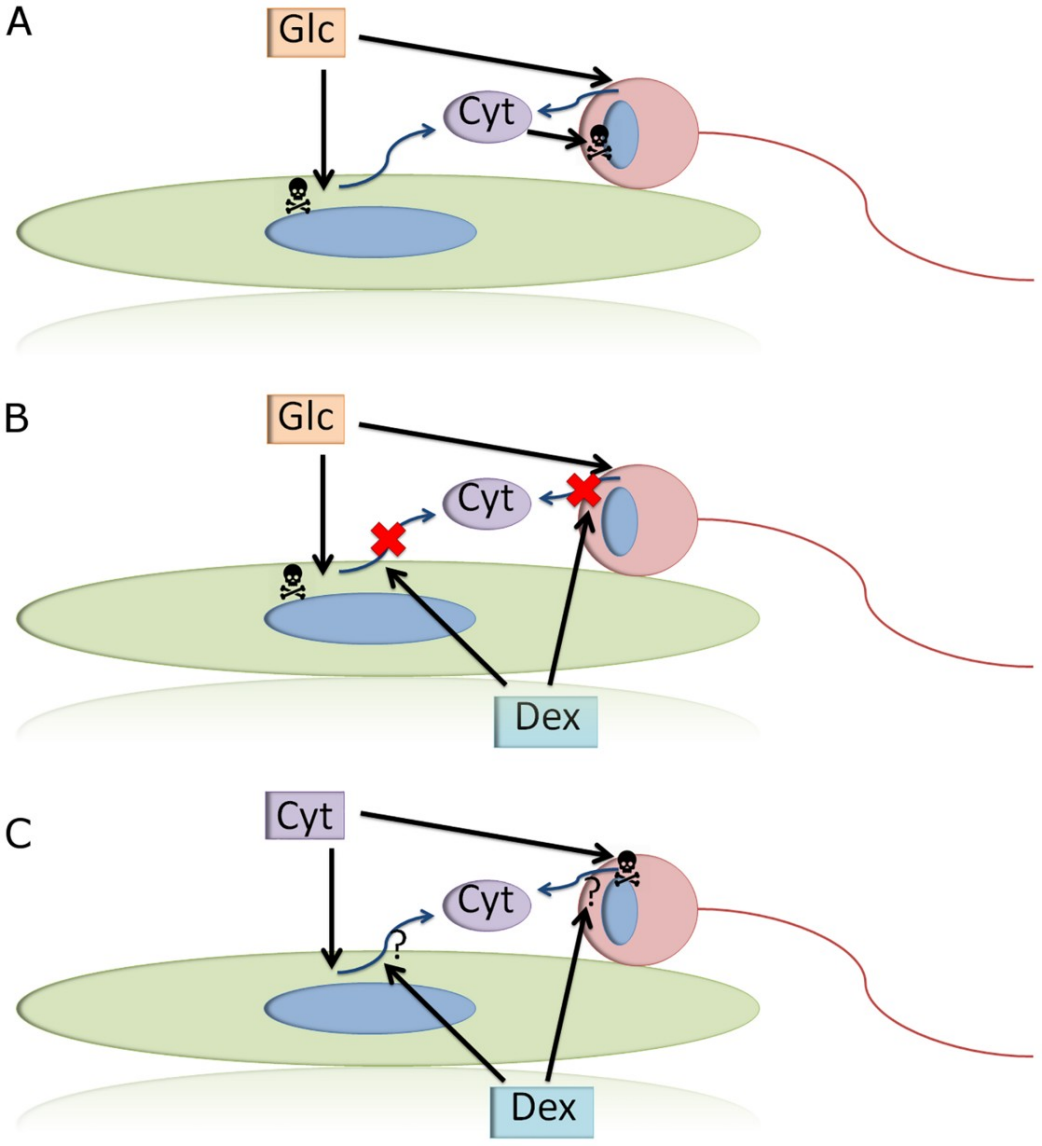
Scheme showing the possible mechanisms active in Müller cell and RGC co-cultures exposed to high glucose, dexamethasone and cytokines. (A) High glucose (30 mM) has a detrimental effect on Müller glia (green) and RGCs (red). Cytokine secretion may be responsible for the effects on cell survival observed. (B) When DEX is added in a high glucose environment, the concentration of cytokines decreases and consequently, RGC survival is enhanced. However, Müller cells were not rescued by DEX. (C) When cytokines are added to the media, RGCs survival is compromised while that of Müller cells is not affected, either in the presence or absence of DEX. We conclude that DEX could inhibit cytokine release, and that RGCs and Müller cells may exhibit distinct sensitivity to the action of cytokines. More experiments will be necessary to elucidate the precise mechanism(s) that relate high glucose-induced cytokine secretion and retinal cell survival.

In summary (Fig 5), we conclude that high concentrations of glucose (30 mM) decrease the survival of RGCs when cultured alone or with Müller glia. This effect could be at least partially due to the secretion of cytokines, in particular L-1β, IL-6 and TNFα, which were elevated in the milieu after glucose treatment. The influence of these cytokines was confirmed when they were added exogenously. Moreover, DEX prevents RGC death in hyperglycemic conditions, at least partially by dampening the release of cytokines.

## Supporting information

S1 File Cell and cytokine counts. (XLSX)

S2 File Statistical analysis of pure RGC cultures. (DOC)

S3 File Statistical analysis of RGCs in co-culture. (DOC)

S4 File Statistical analysis of Müller cells in co-culture. (DOC)

S5 File Statistical analysis of the multiplex cytokine assay. (DOC)

S6 File Statistical analysis of RGCs in the presence of cytokines. (DOC)

S7 File Statistical analysis of Müller cells in the presence of cytokines. (DOC)
