# Supplementary material for: Dexamethasone protects retinal ganglion cells but not Müller glia against hyperglycemia in vitro
Source: PLoS One. 2018 Nov 26;13(11):e0207913. doi: 10.1371/journal.pone.0207913 (PMC6258116; doi:10.1371/journal.pone.0207913)
Supplement: S2 File — (DOC) [file pone.0207913.s002.doc]

Statistics analysis for RGCs in Pure culture. (Fig. 1)


Control	1	
1uM Dexamethasone	2	
10mM glucose	3	
30mM glucose	4	
30mM glucose+1uM Dexamethasone	5	


Oneway


Notes	
Syntax	ONEWAY RGCs BY Condición
  /STATISTICS HOMOGENEITY
  /MISSING ANALYSIS
  /POSTHOC=TUKEY ALPHA(0.05).	
Resources	Processor Time	00:00:00,00	
	Elapsed Time	00:00:00,02	


[ConjuntoDatos1] 


Test of Homogeneity of Variances	
RGCs  	
Levene Statistic	df1	df2	Sig.	
1,981	4	53	,111	


ANOVA	
RGCs  	
	Sum of Squares	df	Mean Square	F	Sig.	
Between Groups	16362,537	4	4090,634	2,946	,028	
Within Groups	73582,567	53	1388,350			
Total	89945,103	57				


Post Hoc Tests

Multiple Comparisons	
Dependent Variable:   RGCs  	
Tukey HSD  	
(I) Condición	(J) Condición	Mean Difference (I-J)	Std. Error	Sig.	95% Confidence Interval	
					Lower Bound	
1	2	,750	15,212	1,000	-42,21	
	3	15,500	15,212	,846	-27,46	
	4	47,817*	15,954	,032	2,76	
	5	21,583	15,212	,618	-21,37	
2	1	-,750	15,212	1,000	-43,71	
	3	14,750	15,212	,868	-28,21	
	4	47,067*	15,954	,036	2,01	
	5	20,833	15,212	,649	-22,12	
3	1	-15,500	15,212	,846	-58,46	
	2	-14,750	15,212	,868	-57,71	
	4	32,317	15,954	,268	-12,74	
	5	6,083	15,212	,994	-36,87	
4	1	-47,817*	15,954	,032	-92,87	
	2	-47,067*	15,954	,036	-92,12	
	3	-32,317	15,954	,268	-77,37	
	5	-26,233	15,954	,477	-71,29	
5	1	-21,583	15,212	,618	-64,54	
	2	-20,833	15,212	,649	-63,79	
	3	-6,083	15,212	,994	-49,04	
	4	26,233	15,954	,477	-18,82	

Multiple Comparisons	
Dependent Variable:   RGCs  	
Tukey HSD  	
(I) Condición	(J) Condición	95% Confidence Interval	
		Upper Bound	
1	2	43,71	
	3	58,46	
	4	92,87	
	5	64,54	
2	1	42,21	
	3	57,71	
	4	92,12	
	5	63,79	
3	1	27,46	
	2	28,21	
	4	77,37	
	5	49,04	
4	1	-2,76	
	2	-2,01	
	3	12,74	
	5	18,82	
5	1	21,37	
	2	22,12	
	3	36,87	
	4	71,29	

*. The mean difference is significant at the 0.05 level.	


Homogeneous Subsets


RGCs	
Tukey HSDa,b  	
Condición	N	Subset for alpha = 0.05	
		1	2	
4	10	57,60		
5	12	83,83	83,83	
3	12	89,92	89,92	
2	12		104,67	
1	12		105,42	
Sig.		,242	,636	

Means for groups in homogeneous subsets are displayed.	
a. Uses Harmonic Mean Sample Size = 11,538.	
b. The group sizes are unequal. The harmonic mean of the group sizes is used. Type I error levels are not guaranteed.	
