# Supplementary material for: Dexamethasone protects retinal ganglion cells but not Müller glia against hyperglycemia in vitro
Source: PLoS One. 2018 Nov 26;13(11):e0207913. doi: 10.1371/journal.pone.0207913 (PMC6258116; doi:10.1371/journal.pone.0207913)
Supplement: S3 File — (DOC) [file pone.0207913.s003.doc]

Statistics analysis for RGCs in co-cultures. (Fig. 2)


Control	1	
1uM Dexamethasone	2	
10mM glucose	3	
30mM glucose	4	
30mM glucose+1uM Dexamethasone	5	


Oneway


Notes	
Syntax	ONEWAY RGCs BY Condición
  /STATISTICS HOMOGENEITY
  /MISSING ANALYSIS
  /POSTHOC=TUKEY ALPHA(0.05).	
Resources	Processor Time	00:00:00,00	
	Elapsed Time	00:00:00,00	


Test of Homogeneity of Variances	
RGCs  	
Levene Statistic	df1	df2	Sig.	
1,531	4	49	,208	


ANOVA	
RGCs  	
	Sum of Squares	df	Mean Square	F	Sig.	
Between Groups	88236379,870	4	22059094,970	13,535	,000	
Within Groups	79858779,610	49	1629771,012			
Total	168095159,500	53				
Post Hoc Tests


Multiple Comparisons	
Dependent Variable:   RGCs  	
Tukey HSD  	
(I) Condición	(J) Condición	Mean Difference (I-J)	Std. Error	Sig.	95% Confidence Interval	
					Lower Bound	
1	2	-276,883	546,618	,986	-1824,87	
	3	1598,917*	546,618	,040	50,93	
	4	3276,553*	532,893	,000	1767,43	
	5	977,553	532,893	,366	-531,57	
2	1	276,883	546,618	,986	-1271,11	
	3	1875,800*	570,924	,015	258,98	
	4	3553,436*	557,798	,000	1973,79	
	5	1254,436	557,798	,179	-325,21	
3	1	-1598,917*	546,618	,040	-3146,91	
	2	-1875,800*	570,924	,015	-3492,62	
	4	1677,636*	557,798	,032	97,99	
	5	-621,364	557,798	,798	-2201,01	
4	1	-3276,553*	532,893	,000	-4785,68	
	2	-3553,436*	557,798	,000	-5133,09	
	3	-1677,636*	557,798	,032	-3257,29	
	5	-2299,000*	544,355	,001	-3840,58	
5	1	-977,553	532,893	,366	-2486,68	
	2	-1254,436	557,798	,179	-2834,09	
	3	621,364	557,798	,798	-958,29	
	4	2299,000*	544,355	,001	757,42	

Multiple Comparisons	
Dependent Variable:   RGCs  	
Tukey HSD  	
(I) Condición	(J) Condición	95% Confidence Interval	
		Upper Bound	
1	2	1271,11	
	3	3146,91	
	4	4785,68	
	5	2486,68	
2	1	1824,87	
	3	3492,62	
	4	5133,09	
	5	2834,09	
3	1	-50,93	
	2	-258,98	
	4	3257,29	
	5	958,29	
4	1	-1767,43	
	2	-1973,79	
	3	-97,99	
	5	-757,42	
5	1	531,57	
	2	325,21	
	3	2201,01	
	4	3840,58	

*. The mean difference is significant at the 0.05 level.	


Homogeneous Subsets


RGCs	
Tukey HSDa,b  	
Condición	N	Subset for alpha = 0.05	
		1	2	3	
4	11	1621,36			
3	10		3299,00		
5	11		3920,36	3920,36	
1	12			4897,92	
2	10			5174,80	
Sig.		1,000	,791	,169	

Means for groups in homogeneous subsets are displayed.	
a. Uses Harmonic Mean Sample Size = 10,749.	
b. The group sizes are unequal. The harmonic mean of the group sizes is used. Type I error levels are not guaranteed.	
