# Supplementary material for: Dexamethasone protects retinal ganglion cells but not Müller glia against hyperglycemia in vitro
Source: PLoS One. 2018 Nov 26;13(11):e0207913. doi: 10.1371/journal.pone.0207913 (PMC6258116; doi:10.1371/journal.pone.0207913)
Supplement: S4 File — (DOC) [file pone.0207913.s004.doc]

Statistics analysis for Müller cells in co-cultures. (Fig. 2)

Control	1	
1uM Dexamethasone	2	
10mM glucose	3	
30mM glucose	4	
30mM glucose+1uM Dexamethasone	5	


Oneway

Notes	
Syntax	ONEWAY Müller BY Condición
  /STATISTICS HOMOGENEITY
  /MISSING ANALYSIS
  /POSTHOC=GH ALPHA(0.05).	
Resources	Processor Time	00:00:00,00	
	Elapsed Time	00:00:00,00	


Test of Homogeneity of Variances	
Müller  	
Levene Statistic	df1	df2	Sig.	
3,604	4	42	,013	


ANOVA	
Müller  	
	Sum of Squares	df	Mean Square	F	Sig.	
Between Groups	10991041070,000	4	2747760268,000	22,847	,000	
Within Groups	5051334115,000	42	120269859,900			
Total	16042375190,000	46				


Post Hoc Tests


Multiple Comparisons	
Dependent Variable:   Müller  	
Games-Howell  	
(I) Condición	(J) Condición	Mean Difference (I-J)	Std. Error	Sig.	95% Confidence Interval	
					Lower Bound	
1	2	10158,533	5574,908	,408	-7816,79	
	3	35452,950*	4623,276	,000	20590,68	
	4	38636,000*	3268,839	,000	28751,55	
	5	30328,800*	4479,500	,000	16460,79	
2	1	-10158,533	5574,908	,408	-28133,86	
	3	25294,417*	6472,615	,011	5238,30	
	4	28477,467*	5586,177	,002	10485,33	
	5	20170,267*	6370,713	,042	550,36	
3	1	-35452,950*	4623,276	,000	-50315,22	
	2	-25294,417*	6472,615	,011	-45350,53	
	4	3183,050	4636,858	,956	-11702,88	
	5	-5124,150	5557,073	,884	-22206,57	
4	1	-38636,000*	3268,839	,000	-48520,45	
	2	-28477,467*	5586,177	,002	-46469,61	
	3	-3183,050	4636,858	,956	-18068,98	
	5	-8307,200	4493,516	,384	-22205,98	
5	1	-30328,800*	4479,500	,000	-44196,81	
	2	-20170,267*	6370,713	,042	-39790,18	
	3	5124,150	5557,073	,884	-11958,27	
	4	8307,200	4493,516	,384	-5591,58	

Multiple Comparisons	
Dependent Variable:   Müller  	
Games-Howell  	
(I) Condición	(J) Condición	95% Confidence Interval	
		Upper Bound	
1	2	28133,86	
	3	50315,22	
	4	48520,45	
	5	44196,81	
2	1	7816,79	
	3	45350,53	
	4	46469,61	
	5	39790,18	
3	1	-20590,68	
	2	-5238,30	
	4	18068,98	
	5	11958,27	
4	1	-28751,55	
	2	-10485,33	
	3	11702,88	
	5	5591,58	
5	1	-16460,79	
	2	-550,36	
	3	22206,57	
	4	22205,98	

*. The mean difference is significant at the 0.05 level.	
