# Supplementary material for: Dexamethasone protects retinal ganglion cells but not Müller glia against hyperglycemia in vitro
Source: PLoS One. 2018 Nov 26;13(11):e0207913. doi: 10.1371/journal.pone.0207913 (PMC6258116; doi:10.1371/journal.pone.0207913)
Supplement: S5 File — (DOC) [file pone.0207913.s005.doc]

Statistics analysis for Multiplex cytokine assay in co-cultures. (Fig. 3)

Control	1	
1uM Dexamethasone	2	
10mM glucose	3	
30mM glucose	4	
30mM glucose+1uM Dexamethasone	5	


Oneway


Notes	
Syntax	ONEWAY IL1b IL6 TNFa BY Condición
  /STATISTICS HOMOGENEITY
  /MISSING ANALYSIS.	
Resources	Processor Time	00:00:00,00	
	Elapsed Time	00:00:00,00	


Oneway


Notes	
Syntax	ONEWAY IL1b IL6 BY Condición
  /STATISTICS HOMOGENEITY
  /MISSING ANALYSIS
  /POSTHOC=GH ALPHA(0.05).	
Resources	Processor Time	00:00:00,02	
	Elapsed Time	00:00:00,01	


Test of Homogeneity of Variances	
	Levene Statistic	df1	df2	Sig.	
IL1b	49,597	4	15	,000	
IL6	3,887	4	15	,023	


ANOVA	
	Sum of Squares	df	Mean Square	F	Sig.	
IL1b	Between Groups	12259,627	4	3064,907	56,439	,000	
	Within Groups	814,565	15	54,304			
	Total	13074,191	19				
IL6	Between Groups	4583680,654	4	1145920,164	17,634	,000	
	Within Groups	974771,305	15	64984,754			
	Total	5558451,959	19				


Post Hoc Tests


Multiple Comparisons	
Games-Howell  	
Dependent Variable	(I) Condición	(J) Condición	Mean Difference (I-J)	Std. Error	Sig.	95% Confidence Interval	
						Lower Bound	Upper Bound	
IL1b	1	2	,000000000000000	,000000000000000	.	,000000000000000	,000000000000000	
		3	,000000000000000	,000000000000000	.	,000000000000000	,000000000000000	
		4	-61,896149940000000*	8,238955595000000	,019	-105,599552199999990	-18,192747720000000	
		5	,000000000000000	,000000000000000	.	,000000000000000	,000000000000000	
	2	1	,000000000000000	,000000000000000	.	,000000000000000	,000000000000000	
		3	,000000000000000	,000000000000000	.	,000000000000000	,000000000000000	
		4	-61,896149940000000*	8,238955595000000	,019	-105,599552199999990	-18,192747720000000	
		5	,000000000000000	,000000000000000	.	,000000000000000	,000000000000000	
	3	1	,000000000000000	,000000000000000	.	,000000000000000	,000000000000000	
		2	,000000000000000	,000000000000000	.	,000000000000000	,000000000000000	
		4	-61,896149940000000*	8,238955595000000	,019	-105,599552199999990	-18,192747720000000	
		5	,000000000000000	,000000000000000	.	,000000000000000	,000000000000000	
	4	1	61,896149940000000*	8,238955595000000	,019	18,192747720000000	105,599552199999990	
		2	61,896149940000000*	8,238955595000000	,019	18,192747720000000	105,599552199999990	
		3	61,896149940000000*	8,238955595000000	,019	18,192747720000000	105,599552199999990	
		5	61,896149940000000*	8,238955595000000	,019	18,192747720000000	105,599552199999990	
	5	1	,000000000000000	,000000000000000	.	,000000000000000	,000000000000000	
		2	,000000000000000	,000000000000000	.	,000000000000000	,000000000000000	
		3	,000000000000000	,000000000000000	.	,000000000000000	,000000000000000	
		4	-61,896149940000000*	8,238955595000000	,019	-105,599552199999990	-18,192747720000000	
IL6	1	2	410,816924699999960*	55,567429920000000	,003	193,271458000000000	628,362391500000000	
		3	-174,872984500000000	83,153326310000000	,365	-545,628417700000000	195,882448700000000	
		4	-1019,488323999999900	252,866349300000000	,098	-2331,714322999999700	292,737675700000000	
		5	79,563684400000000	101,307350200000000	,922	-395,113849999999960	554,241218800000000	
	2	1	-410,816924699999960*	55,567429920000000	,003	-628,362391500000000	-193,271458000000000	
		3	-585,689909200000000*	89,545071400000000	,007	-948,294910200000000	-223,084908300000000	
		4	-1430,305248000000000*	255,039672900000000	,038	-2724,045374000000000	-136,565123200000000	
		5	-331,253240299999960	106,616243300000000	,139	-788,902298600000000	126,395817900000000	
	3	1	174,872984500000000	83,153326310000000	,365	-195,882448700000000	545,628417700000000	
		2	585,689909200000000*	89,545071400000000	,007	223,084908300000000	948,294910200000000	
		4	-844,615339200000000	262,434698800000000	,149	-2089,395602000000000	400,164924000000000	
		5	254,436668900000000	123,262969899999990	,345	-215,488034600000000	724,361372500000000	
	4	1	1019,488323999999900	252,866349300000000	,098	-292,737675700000000	2331,714322999999700	
		2	1430,305248000000000*	255,039672900000000	,038	136,565123200000000	2724,045374000000000	
		3	844,615339200000000	262,434698800000000	,149	-400,164924000000000	2089,395602000000000	
		5	1099,052008000000000	268,739045699999960	,069	-117,606543500000000	2315,710560000000000	
	5	1	-79,563684400000000	101,307350200000000	,922	-554,241218800000000	395,113849999999960	
		2	331,253240299999960	106,616243300000000	,139	-126,395817900000000	788,902298600000000	
		3	-254,436668900000000	123,262969899999990	,345	-724,361372500000000	215,488034600000000	
		4	-1099,052008000000000	268,739045699999960	,069	-2315,710560000000000	117,606543500000000	

*. The mean difference is significant at the 0.05 level.	


Oneway


Notes	
Syntax	ONEWAY TNFa BY Condición
  /STATISTICS HOMOGENEITY
  /MISSING ANALYSIS
  /POSTHOC=TUKEY ALPHA(0.05).	
Resources	Processor Time	00:00:00,02	
	Elapsed Time	00:00:00,02	


Test of Homogeneity of Variances	
TNFa  	
Levene Statistic	df1	df2	Sig.	
2,510	4	15	,086	


ANOVA	
TNFa  	
	Sum of Squares	df	Mean Square	F	Sig.	
Between Groups	20191,879	4	5047,970	15,463	,000	
Within Groups	4896,793	15	326,453			
Total	25088,672	19				


Post Hoc Tests


Multiple Comparisons	
Dependent Variable:   TNFa  	
Tukey HSD  	
(I) Condición	(J) Condición	Mean Difference (I-J)	Std. Error	Sig.	95% Confidence Interval	
					Lower Bound	
1	2	46,775696990000000*	12,776010060000000	,017	7,324342633000001	
	3	3,055492848000000	12,776010060000000	,999	-36,395861510000000	
	4	-53,012254460000000*	12,776010060000000	,007	-92,463608820000000	
	5	6,527517009000000	12,776010060000000	,985	-32,923837350000000	
2	1	-46,775696990000000*	12,776010060000000	,017	-86,227051350000000	
	3	-43,720204140000000*	12,776010060000000	,027	-83,171558500000000	
	4	-99,787951450000010*	12,776010060000000	,000	-139,239305799999980	
	5	-40,248179980000000*	12,776010060000000	,045	-79,699534340000000	
3	1	-3,055492848000000	12,776010060000000	,999	-42,506847210000004	
	2	43,720204140000000*	12,776010060000000	,027	4,268849785000001	
	4	-56,067747310000000*	12,776010060000000	,004	-95,519101660000000	
	5	3,472024161000000	12,776010060000000	,999	-35,979330200000000	
4	1	53,012254460000000*	12,776010060000000	,007	13,560900100000000	
	2	99,787951450000010*	12,776010060000000	,000	60,336597090000000	
	3	56,067747310000000*	12,776010060000000	,004	16,616392950000000	
	5	59,539771470000000*	12,776010060000000	,002	20,088417110000000	
5	1	-6,527517009000000	12,776010060000000	,985	-45,978871370000000	
	2	40,248179980000000*	12,776010060000000	,045	,796825624000000	
	3	-3,472024161000000	12,776010060000000	,999	-42,923378520000000	
	4	-59,539771470000000*	12,776010060000000	,002	-98,991125830000000	

Multiple Comparisons	
Dependent Variable:   TNFa  	
Tukey HSD  	
(I) Condición	(J) Condición	95% Confidence Interval	
		Upper Bound	
1	2	86,227051350000000	
	3	42,506847210000004	
	4	-13,560900100000000	
	5	45,978871370000000	
2	1	-7,324342633000001	
	3	-4,268849785000001	
	4	-60,336597090000000	
	5	-,796825624000000	
3	1	36,395861510000000	
	2	83,171558500000000	
	4	-16,616392950000000	
	5	42,923378520000000	
4	1	92,463608820000000	
	2	139,239305799999980	
	3	95,519101660000000	
	5	98,991125830000000	
5	1	32,923837350000000	
	2	79,699534340000000	
	3	35,979330200000000	
	4	-20,088417110000000	

*. The mean difference is significant at the 0.05 level.	


Homogeneous Subsets


TNFa	
Tukey HSDa  	
Condición	N	Subset for alpha = 0.05	
		1	2	3	
2	4	18,830938880000000			
5	4		59,079118860000000		
3	4		62,551143020000000		
1	4		65,606635870000000		
4	4			118,618890299999990	
Sig.		1,000	,985	1,000	

Means for groups in homogeneous subsets are displayed.	
a. Uses Harmonic Mean Sample Size = 4,000.	
