# Supplementary material for: Dexamethasone protects retinal ganglion cells but not Müller glia against hyperglycemia in vitro
Source: PLoS One. 2018 Nov 26;13(11):e0207913. doi: 10.1371/journal.pone.0207913 (PMC6258116; doi:10.1371/journal.pone.0207913)
Supplement: S6 File — (DOC) [file pone.0207913.s006.doc]

Statistics analysis for RGCs in Cytokines experiment. (Fig. 4)


Control	1	
1uM Dex	2	
IL-1â, IL-6, TNFá (ng/ml) 	3	
IL-1â, IL-6, TNFá (pg/ml) 	4	
IL-1â, IL-6, TNFá (ng/ml) + 1µM Dex	5	
IL-1â, IL-6, TNFá (pg/ml) + 1µM Dex	6	


Oneway

Notes	
Syntax	ONEWAY RGCs BY Condición
  /STATISTICS HOMOGENEITY
  /MISSING ANALYSIS
  /POSTHOC=TUKEY ALPHA(0.05).	
Resources	Processor Time	00:00:00,00	
	Elapsed Time	00:00:00,00	


Test of Homogeneity of Variances	
RGCs  	
Levene Statistic	df1	df2	Sig.	
1,323	5	18	,299	


ANOVA	
RGCs  	
	Sum of Squares	df	Mean Square	F	Sig.	
Between Groups	21154270,660	5	4230854,132	8,541	,000	
Within Groups	8916311,300	18	495350,628			
Total	30070581,960	23				


Post Hoc Tests

Multiple Comparisons	
Dependent Variable:   RGCs  	
Tukey HSD  	
(I) Condición	(J) Condición	Mean Difference (I-J)	Std. Error	Sig.	95% Confidence Interval	
					Lower Bound	Upper Bound	
1	2	452,200	513,991	,947	-1181,28	2085,68	
	3	2441,750*	537,545	,003	733,41	4150,09	
	4	1795,500*	537,545	,036	87,16	3503,84	
	5	2167,000*	537,545	,009	458,66	3875,34	
	6	2413,250*	537,545	,003	704,91	4121,59	
2	1	-452,200	513,991	,947	-2085,68	1181,28	
	3	1989,550*	472,131	,006	489,10	3490,00	
	4	1343,300	472,131	,095	-157,15	2843,75	
	5	1714,800*	472,131	,020	214,35	3215,25	
	6	1961,050*	472,131	,007	460,60	3461,50	
3	1	-2441,750*	537,545	,003	-4150,09	-733,41	
	2	-1989,550*	472,131	,006	-3490,00	-489,10	
	4	-646,250	497,670	,782	-2227,86	935,36	
	5	-274,750	497,670	,993	-1856,36	1306,86	
	6	-28,500	497,670	1,000	-1610,11	1553,11	
4	1	-1795,500*	537,545	,036	-3503,84	-87,16	
	2	-1343,300	472,131	,095	-2843,75	157,15	
	3	646,250	497,670	,782	-935,36	2227,86	
	5	371,500	497,670	,973	-1210,11	1953,11	
	6	617,750	497,670	,811	-963,86	2199,36	
5	1	-2167,000*	537,545	,009	-3875,34	-458,66	
	2	-1714,800*	472,131	,020	-3215,25	-214,35	
	3	274,750	497,670	,993	-1306,86	1856,36	
	4	-371,500	497,670	,973	-1953,11	1210,11	
	6	246,250	497,670	,996	-1335,36	1827,86	
6	1	-2413,250*	537,545	,003	-4121,59	-704,91	
	2	-1961,050*	472,131	,007	-3461,50	-460,60	
	3	28,500	497,670	1,000	-1553,11	1610,11	
	4	-617,750	497,670	,811	-2199,36	963,86	
	5	-246,250	497,670	,996	-1827,86	1335,36	

*. The mean difference is significant at the 0.05 level.	


Homogeneous Subsets


RGCs	
Tukey HSDa,b  	
Condición	N	Subset for alpha = 0.05	
		1	2	3	
3	4	939,25			
6	4	967,75			
5	4	1214,00			
4	4	1585,50	1585,50		
2	5		2928,80	2928,80	
1	3			3381,00	
Sig.		,789	,131	,942	

Means for groups in homogeneous subsets are displayed.	
a. Uses Harmonic Mean Sample Size = 3,913.	
b. The group sizes are unequal. The harmonic mean of the group sizes is used. Type I error levels are not guaranteed.	
