# Supplementary material for: Dexamethasone protects retinal ganglion cells but not Müller glia against hyperglycemia in vitro
Source: PLoS One. 2018 Nov 26;13(11):e0207913. doi: 10.1371/journal.pone.0207913 (PMC6258116; doi:10.1371/journal.pone.0207913)
Supplement: S7 File — (DOC) [file pone.0207913.s007.doc]

Statistics analysis for Müller cells in Cytokines experiment. (Fig. 4)


Control	1	
1uM Dex	2	
IL-1â, IL-6, TNFá (ng/ml) 	3	
IL-1â, IL-6, TNFá (pg/ml) 	4	
IL-1â, IL-6, TNFá (ng/ml) + 1µM Dex	5	
IL-1â, IL-6, TNFá (pg/ml) + 1µM Dex	6	


Oneway

Notes	
Syntax	ONEWAY Müller BY Condición
  /STATISTICS HOMOGENEITY
  /MISSING ANALYSIS.	
Resources	Processor Time	00:00:00,02	
	Elapsed Time	00:00:00,02	

Test of Homogeneity of Variances	
Müller  	
Levene Statistic	df1	df2	Sig.	
,466	5	15	,796	


ANOVA	
Müller  	
	Sum of Squares	df	Mean Square	F	Sig.	
Between Groups	577887548,000	5	115577509,600	,164	,972	
Within Groups	10571321600,000	15	704754773,400			
Total	11149209150,000	20				
